# Automated Region of Interest Selection Improves Deep Learning-Based Segmentation of Hyper-Reflective Foci in Optical Coherence Tomography Images

**DOI:** 10.3390/jcm11247404

**Published:** 2022-12-14

**Authors:** Sarang Goel, Abhishek Sethi, Maximilian Pfau, Monique Munro, Robison Vernon Paul Chan, Jennifer I. Lim, Joelle Hallak, Minhaj Alam

**Affiliations:** 1 Texas Academy of Mathematics and Science, Denton, TX 76203, USA; 2 Department of Ophthalmology and Visual Sciences, University of Illinois at Chicago, Chicago, IL 60612, USA; 3 Institute of Molecular and Clinical Ophthalmology Basel, 4031 Basel, Switzerland; 4 Department of Electrical and Computer Engineering, University of North Carolina at Charlotte, Charlotte, NC 28223, USA

**Keywords:** hyperreflective foci, deep learning, segmentation, ophthalmic AI, diabetic retinopathy, age-related macular degeneration

## Abstract

Hyperreflective foci (HRF) have been associated with retinal disease progression and demonstrated as a negative prognostic biomarker for visual function. Automated segmentation of HRF in retinal optical coherence tomography (OCT) scans can be beneficial to identify the formation and movement of the HRF biomarker as a retinal disease progresses and can serve as the first step in understanding the nature and severity of the disease. In this paper, we propose a fully automated deep neural network based HRF segmentation model in OCT images. We enhance the model’s performance by using a patch-based strategy that increases the model’s compute on the HRF pixels. The patch-based strategy is evaluated against state of the art HRF segmentation pipelines on clinical retinal image data. Our results shows that the patch-based approach demonstrates a high precision score and intersection over union (IOU) using a ResNet34 segmentation model with Binary Cross Entropy loss function. The HRF segmentation pipeline can be used for analyzing HRF biomarkers for different retinopathies.

## 1. Introduction

Hyperreflective foci (HRF) are small, well-circumscribed, dot-shaped lesions with equal or greater reflectivity than the retinal pigment epithelium (RPE) band on retinal optical coherence tomography (OCT) scans [1,2,3,4]. HRF have been linked with numerous retinal diseases, including diabetic retinopathy (DR) and age-related macular degeneration (AMD) [5], often targeting the elderly population [6] and leading to visual impairment. Past studies demonstrate the use of HRF as a biomarker to track the progressive state of retinal tissue inflammation caused by retinal diseases, such as AMD [7,8,9]. HRF has also been demonstrated to have a negative correlation with visual function [9]. The segmentation of HRF in retinal OCT scans for the purpose of identifying the formation and movement of the biomarker along the course of retinal diseases can serve as the first step in understanding the nature and severity of the disease for a particular patient. However, manual segmentation of HRF in retinal OCT volumes, which consists of hundreds of B-Scan sections, is a very tedious and error-prone process. On the other hand, automated segmentation provides a fast and accurate analysis of HRF.

There have been studies that have attempted to segment HRF manually, semi-manually or automatically using image processing and machine learning (ML)/deep learning (DL) methodologies [10,11,12,13,14,15]. In a manual analysis of HRF in OCT scans, a correlation between HRF and AMD was found in [10]. Another study focused on the semi-manual analysis of HRF in OCT scans, where a semi-automated algorithm was created to quantify HRF in OCT scans and evaluated based on the results of successive OCT scans from the same patient [10]. Neither of these methods of analysis involved the use of machine learning methods, or the use of pixel-level ground truth data for the evaluation of the algorithm. Additionally, other studies have proposed algorithms to conduct automatic segmentation of HRF in OCT scans [11,12,13,14,15]. However, the algorithms described in these studies require a large processing time, lack a generalized approach for thresholding, and can be prone to noise from the OCT images. Furthermore, for DL based segmentation, using the whole OCT image for segmentation can lead to lower accuracy since the model is trained only on a small ratio of HRF pixels (in patches near the outer retinal layers), and DL segmentation models need to be trained on a high ratio of such pixels so they can learn their characteristics and perform well. In this paper, we propose a deep neural network to automatically segment HRF in retinal OCT scans using a patch-based approach. The image patches were extracted from OCT B-Scans and consisted of >90% retinal pixels (background was removed). We compared the patch-based approach with baseline segmentation networks trained in whole OCT B-Scans and cropped OCT B-Scans (showing only retinal pixels). We performed an evaluation of different training backbones, training loss, augmentations, and pre-processing techniques for the retinal OCT scans (full image, cropped image, and patches) to improve the segmentation training. The model was trained, validated, and tested on real-life clinical data. Our segmentation analysis on th HRFs showed that the patch-based approach, which increased the compute on more HRF pixels, provided the best segmentation accuracy, even on data that the model had not seen before.

## 2. Materials and Methods

### 2.1. Data Collection

**Figure 1:**
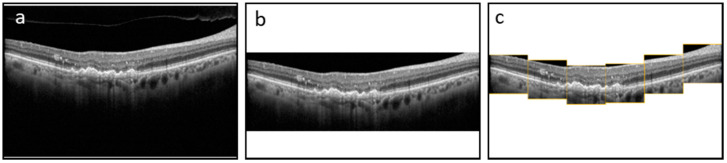
Three training image types—(**a**) Full image; (**b**) Cropped image; (**c**) Patches.

The dataset consists of 3557 B-Scans and their corresponding ground truth (GT) masks collected from 600 DR and 2957 AMD subjects at a tertiary center at the University Eye Hospital Bonn, University of Bonn, Germany. Spectral-domain OCT imaging was performed using a Spectralis HRA+OCT (digital image resolution, 496 × 768 pixels; Heidelberg Engineering, Heidelberg, Germany) device, through the fovea. A minimum signal strength of 7 out of 10 was considered for the inclusion of data. Data was de-identified and exported in an anonymized manner. This study adhered to the tenets of the Declaration of Helsinki and was approved by the local institutional review boards at the University of Bonn (Bonn) and the University of Illinois at Chicago (UIC). The GT was generated by one grader at Bonn (MP) and two graders at UIC (MM and AS—supervised by MM). ImageJ was used to manually annotate the boundaries of HRFs found in the outer nuclear and/or outer plexiform layers for each OCT image. We measured the variability of the manual segmentation of HRFs between two sets of graders and evaluated the accuracy of the DL model compared to manual segmentation by both teams. Each of the B-Scans contained at least one HRF. The size of the B-Scans and masks are 496 × 768 pixels. The original dataset was sorted and shuffled with a random state and split 80/20 into a train and test dataset. Furthermore, the train dataset was split 80/20 to create a validation dataset. The final train, validation, and test datasets consisted of 2276, 569, and 217 B-Scans and their corresponding GT masks, respectively. We trained and evaluated the segmentation model using three schemes: with full B-Scan images, cropped B-Scan images, and patches of B-Scan images, as shown in Figure 1.

### 2.2. Preprocessing of OCT Images for HRF Segmentation

A pretrained layer segmentation model was applied on each B-Scan in the train and validation datasets [16]. The segmentation model was a convolutional neural network (CNN; DeeplabV3 with ResNet-50 backbone [17]) previously trained on 9680 B-Scans from 80 patients with late AMD (40 patients with macular neovascularization (MNV) and 40 patients with geographic atrophy (GA)). The final model was selected on the basis of an optimal validation loss (to avoid overfitting) and then an inference model was used on our current HRF OCT data. From the qualitative observations, the majority of the hyper-reflective foci were located in the outer retina around the photoreceptor layers and outer nuclear layer (ONL).

The model identifies six different layers present in each B-Scan. Using the output layers, sixty-three 128 × 128 pixel patches were extracted for every B-Scan using the following procedure:

1. The first patch was placed at the left edge of the B-Scan (x-coordinates of 0–127) and was vertically aligned on the 3rd layer (IS) found in the patch area.

2. The next patch was horizontally straddled by 25% of the patch size (32 pixels) to the right and was vertically centered on the same layer found in the patch area.

3. Patches continued to be straddled until the last patch reached the right edge of the B-Scan. This resulted in 21 patches across the width of the B-Scan.

4. Next, each of the 21 patches was straddled vertically up and vertically down by 25% of the patch size (32 pixels). This resulted in 21 × 3 = 63 patches per B-Scan.

5. Corresponding patches were taken from the GT mask to produce 63 patches per GT mask as well.

Through this procedure, the total number of patches generated from the train and validation datasets amounted to 63 × 2845 = 179,235 B-Scan and GT mask patches. Finally, the GT mask patches and corresponding B-Scan patches were separated into HRF and Normal folders, depending on the max pixel value of each GT mask patch (where greater than 0 signifies HRF, while equal to 0 signifies Normal). The total number of B-Scan and GT mask patches labeled as HRF and Normal were 45,080 and 126,162, respectively.

### 2.3. Classification Model for Patches

**Figure 2:**
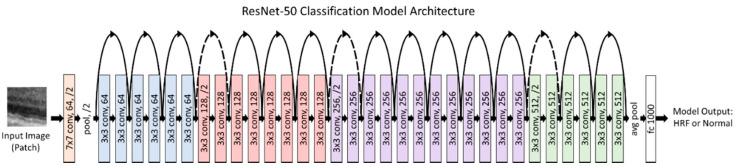
ResNet50 model for HRF patch classification.

When the model is trained on patches, an initial step of classifying whether the patch has HRF in it or not is required prior to training. This is accomplished by using a custom classification model, which was evaluated for different models (VGG-16, ResNet50, InceptionV3, and EfficientNetB0 [18,19,20]). The purpose of the first classification model was to identify whether a patch had HRF or not. Therefore, we had two prediction classes: HRF and Normal. The B-Scan patches from the train and validation dataset were used to train and validate our model. Our classification model was trained using a batch size of 256 and a learning rate of 10^−4^ and was trained for 50 epochs. Additionally, the patches were augmented to ensure a robust model. These augmentations included a rotation of 0–10 degrees, a brightness range of 0.8 to 1.2, and a horizontal flip. The classification model used transfer learning with ImageNet weights to reach rapid progress in training and achieve a high performance. Figure 2 illustrates our final classification model architecture.

### 2.4. Segmentation Model for Patches

The purpose of the segmentation model was to segment the location of the HRF in the B-Scan. The B-Scan patches from the train and validation dataset that were labeled as HRF were used to train and validate our model. Our segmentation model was trained using a batch size of 10 (small due to memory limitations) and a learning rate of 10^−4^ (small as it allows the model to learn more optimally) and was trained for 1500 epochs. The segmentation model used the U-Net architecture and was tested on multiple image types as shown in Figure 1. Additionally, we conducted rigorous hypertuning, including testing different loss functions and backbones in order to achieve the highest performance. The multiple loss functions we evaluated included dice loss, Jaccard loss, and Binary Cross Entropy. Additionally, the multiple backbones we evaluated included ResNet34, InceptionV3, DenseNet169, and EfficientNetB4 with augmentations such as dilation, rotation, brightness, and horizontal flip.

**Figure 3:**
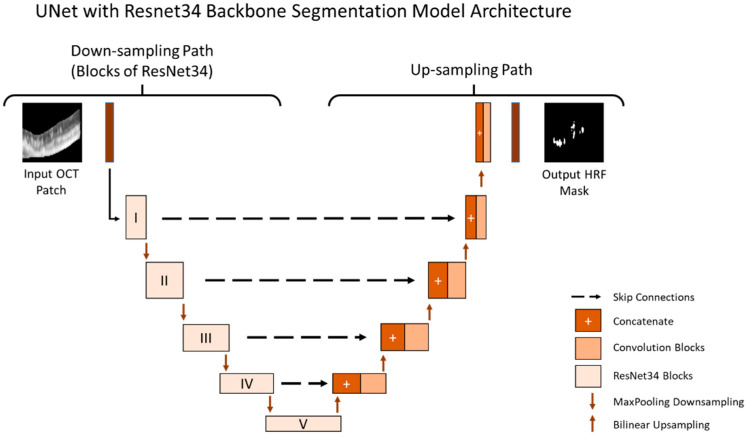
U-Net segmentation model for HRF segmentation.

All models were trained and stored at the epoch with the best performance on the validation set. The final performance for all models were evaluated on the test set. The final segmentation model is illustrated in Figure 3.

### 2.5. Applying the Classification and Segmentation Model on the Test Dataset and Outcome Evaluation Criteria

For the final performance of the model using patches, a pretrained layer segmentation model was applied on each B-Scan in the test dataset. The model identified six different layers present in each B-Scan. Using the output layers, six 128 × 128 pixel patches were extracted in real-time for each B-Scan using the following procedure:

1. The first patch was placed at the left edge of the B-Scan (x-coordinates of 0–127) and was vertically aligned on the 3rd layer (IS) found in the patch area.

2. The next patch was horizontally straddled by 100% of the patch size (128 pixels) to the right and was vertically centered on the same layer found in the patch area.

3. Patches continued to be straddled until the last patch reached the right edge of the B-Scan. This resulted in 6 patches across the width of the B-Scan (6 × 128 = 768 W pixel).

**Figure 4:**
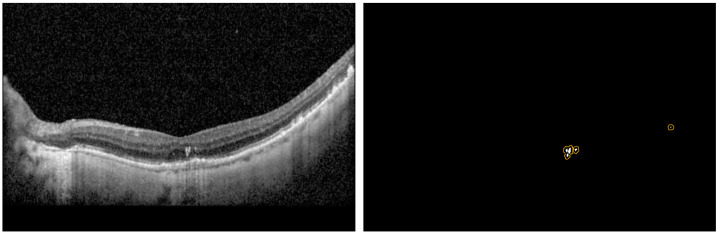
Visualization of prediction with nxn windowing.

Each patch was then classified as HRF or Normal using the finalized classification model. The segmentation model was then used for segmenting the location of the HRF in the patches classified as HRF. The 6 patches were stitched back into a blank image of the same size of the original B-Scan to create the predicted mask, and thus obtain the intersection over union (IOU) and Precision per B-Scan. The analysis was completed for the entire test dataset to determine the average performance metrics of the IOU, Dice, and Precision scores. Moreover, we came up with three new metrics referred to as modified_IOU, modified_Dice, and modified_Precision. We identified nxn (n = 0, 2, 4, 8) pixel window centered around the predicted pixel to see if a ground truth pixel was present. Figure 4 shows an example of the predicted pixels (in white) and the surrounding boundary (in orange) indicating the extended HRF region in the vicinity of the prediction.

## 3. Results

We evaluated multiple common pre-trained models, including VGG-16, ResNet50, InceptionV3, and EfficientNetB0, and found that ResNet50 provided the best accuracy to identify HRFs in a patch. Table 1 shows the classification results for the various models we tested.

We also evaluated multiple backbones for the U-Net HRF-segmentation models, including ResNet34, InceptionV3, DenseNet169, and EfficientNetB4, and found that ResNet34 provided the best performance. Table 2 shows the segmentation results for the various backbones we tested on cropped images, which had only retinal pixels. The cropped images were the input to the patch-based approach; therefore, the initial screening of the backbones was performed on them.

**Figure 5:**
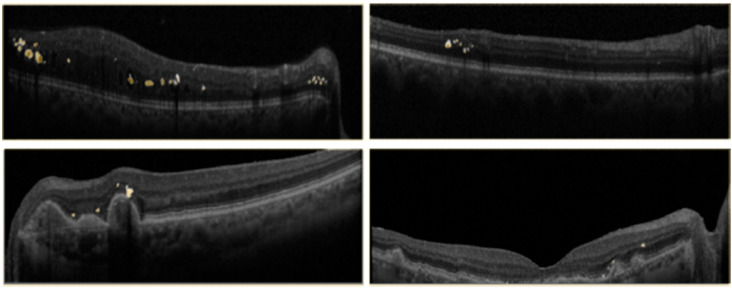
Example HRF segmentation results. Yellow is the prediction over white ground truth annotations.

Table 3 shows the segmentation results for the three image types and loss functions we tested using the U-Net model with the backbone of ResNet34. The best performance for IOU and Precision were obtained with Patches image type and Binary Cross Entropy loss function. Some examples of the segmentation prediction are shown in Figure 5.

Table 4 shows the segmentation results with various window sizes, including 0 × 0 (no window), 2 × 2, 4 × 4, and 8 × 8 during the patch extraction.

## 4. Discussion

In this study, we implemented a deep neural network to automatically segment HRF in retinal OCT scans. We utilized a U-Net for accurate HRF segmentation. We performed an evaluation of the different training backbones (ResNet34, Inceptionv3, Densenet169, and Efficientnetb4) and observed that the ResNet34 backbone demonstrated the best results. Next, we evaluated multiple training loss functions and observed that the best choice of loss function varied with the pre-processing technique (full image, cropped image, and patches) employed to improve segmentation training. When using the full image, all three loss functions gave similar results. When using cropped images, both Dice loss and Jaccard loss gave better results. When using patches, Binary Cross Entropy gave the best results with an IOU of 0.392, Dice score of 0.502, and Precision of 0.70.

To generate the patches, we also utilized a pretrained layer segmentation model (centered patches around retinal layers with high HRF concentration). When the model is trained on patches, an initial step of classifying whether the patch has HRF in it or not is required prior to training. This is done to help increase the ratio of HRF pixels in the patches during the training and validation. Without this step, it would result in a low average precision score for the model as deep learning segmentation models need to be trained on a high ratio of pixels of interests (i.e., HRF pixels) so they can learn their characteristics and perform well. This is accomplished by using a custom classification model, which was evaluated for different models (VGG-16, ResNet50, InceptionV3, and EfficientNetB0) and Categorical Cross Entropy loss function. ResNet50 gave the best results with an Accuracy of 0.905, AUC score of 0.849, HRF Patch Precision/Recall of 0.873/0.736, and Normal Patch Precision/Recall of 0.911/0.962.

Finally, we observed that while the IOU scores were not very high, the HRF predicted pixel locations were still in the same areas of the B-Scan ground truth pixels. We believe that this would still be very helpful for the physicians who are interested in locating HRFs in the retina. We came up with a method to quantify this benefit using new metrics referred to as modified_IOU, modified_Dice, and modified_Precision. We identified a nxn pixel window centered around the predicted pixel to see if a ground truth pixel was present. Using a U-Net model with ResNet34 backbone and Binary Cross Entropy loss function, we observed that as we increased the window sizes from 0 × 0 (no window) to 2 × 2, 4 × 4, and 8 × 8, the Modified_IOU improved from 0.38 to 0.429, 0.489, and 0.526 respectively; Modified_Dice improved from 0.495 to 0.53, 0.561, and 0.579, respectively; and Modified_Precision improved from 0.681 to 0.728, 0.769, and 0.793, respectively. One thing to note is that, in this study, we employed three different graders to generate the ground truth labels for the segmentation of HRF in retinal OCT scans. The IOU score of the ground truth labels from the three graders was low, suggesting inconsistency and/or inaccuracy in the way the labeling was done. It further highlights the variability and/or difficulty in the task of manually identifying HRF in retinal OCT scans. It is known that HRFs can be characterized with variable traits and are often mis-classified by different observers. Therefore, we believe the model performed decently based on the precision score (0.75—higher than current state of the art), and the modified metrics. One limitation of the study is that the dataset is obtained from only one source that may cause the model to overfit to any peculiarities of the dataset and may not scale well to other sources of dataset. Future studies will be needed to better understand these limitations and improve prediction models.

## 5. Conclusions

In this research, we created a DL model to automatically segment HRF in retinal OCT scans using a patch-based approach. Compared to the state of the art, the DL model introduced in this research achieved a higher average precision. We demonstrated that by providing a higher DL compute to HRF pixels during training (patches with HRFs), we can improve the model’s performance significantly. This automated HRF segmentation pipeline can be used for analyzing HRF biomarkers for different retinopathies.

## Figures and Tables

**Table 1:** Training and testing on B-Scans using (4) models for patch image type.

Image Type	Loss	Model	Accuracy	AUC Score	HRF Patch Precision/Recall	Normal Patch Precision/Recall
Patches	Categorical Cross Entropy	VGG-16	0.828	0.729	0.748/0.52	0.846/0.938
Patches	Categorical Cross Entropy	ResNet50	0.903	0.849	0.873/0.736	0.911/0.962
Patches	Categorical Cross Entropy	InceptionV3	0.796	0.669	0.694/0.401	0.815/0.937
Patches	Categorical Cross Entropy	EfficientNetB0	0.734	0.503	0.354/0.016	0.738/0.99

**Table 2:** Training and testing on B-Scans using (4) model backbones for the cropped image type using Jaccard loss.

Image Type	Loss	Model	Backbone	IOU	Dice	Precision
Cropped Image	Jaccard Loss	U-Net	ResNet34	0.362	0.484	0.554
Cropped Image	Jaccard Loss	U-Net	Inceptionv3	0.344	0.461	0.568
Cropped Image	Jaccard Loss	U-Net	Densenet169	0.293	0.410	0.476
Cropped Image	Jaccard Loss	U-Net	Efficientnetb4	0.356	0.479	0.546

**Table 3:** Training and testing on B-Scans using (3) image types and (3) loss functions.

Image Type	Loss	Model	Backbone	IOU	Dice	Precision
Full Image	Dice Loss	U-Net	ResNet34	0.357	0.483	0.562
	Jaccard Loss	U-Net	ResNet34	0.359	0.484	0.554
	Binary Cross Entropy	U-Net	ResNet34	0.357	0.486	0.586
Cropped Image	Dice Loss	U-Net	ResNet34	0.361	0.49	0.55
	Jaccard Loss	U-Net	Resnet34	0.362	0.484	0.554
	Binary Cross Entropy	U-Net	ResNet34	0.328	0.449	0.515
Patches	Dice Loss	U-Net	ResNet34	0.364	0.489	0.659
	Jaccard Loss	U-Net	Resnet34	0.371	0.494	0.668
	Binary Cross Entropy	U-Net	ResNet34	**0.392**	**0.502**	**0.75**

bold represents the best results obtained in our experiments.

**Table 4:** Training and testing on B-Scans using (4) window sizes for patches using the Binary Cross Entropy loss function and U-Net-ResNet34 model.

Image Type	Loss	Model	Backbone	Window Size	Modified IOU	Modified Dice	Modified Precision
Patches	Binary Cross Entropy	U-Net	ResNet34	0	0.380	0.495	0.681
Patches	Binary Cross Entropy	U-Net	ResNet34	2	0.429	0.530	0.728
Patches	Binary Cross Entropy	U-Net	ResNet34	4	0.489	0.561	0.769
Patches	Binary Cross Entropy	U-Net	ResNet34	8	0.526	0.579	0.793

## Data Availability

Bonn data may be made available upon reasonable request.
